# Association between triglyceride-glucose index and the risk of type 2 diabetes mellitus

**DOI:** 10.20945/2359-4292-2023-0493

**Published:** 2025-04-14

**Authors:** Hui Luo, Qin Yang, Haolan Xu, Shan Wu, Wenjing Wang, Ru Zhou, Yanlang Yang, Qi Yu

**Affiliations:** 1 Department of Nephrology, The First Affiliated Hospital of Wannan Medical College, Yijishan Hospital, Wuhu, Anhui, People’s Republic of China; 2 Nanchang University, Nanchang, Jiangxi, People’s Republic of China

**Keywords:** Triglyceride-glucose index, type 2 diabetes mellitus, insulin resistance, body mass index

## Abstract

**Objective:**

To assess the efficacy of the triglyceride-glucose (TyG) index in predicting type 2 diabetes mellitus (T2DM) in the general population.

**Subjects and methods:**

Baseline data were collected from a community population that underwent physical examination between 2015 and 2020. The TyG index was calculated via the following formula: TyG = Ln [fasting triglyceride (mg/dL) × fasting blood glucose (mg/dL)/2]. Cox regression and stratified analyses were performed to evaluate the ability of the TyG score to predict the occurrence of diabetes.

**Results:**

In total, 8 576 subjects were ultimately included and divided into a T2DM group (n = 882) and a non-T2DM group (n = 7,694) according to the results of the 5-year follow-up. Adjustment for all covariates revealed that every 1-unit increase in the TyG index multiplied the risk of T2DM in all the participants (HR: 3.348; 95% CI: 3.004-3.731; P < 0.001). When TyG was divided into three quantiles, the risk of T2DM in the highest quantile was 6.412 times greater than that in the lowest quantile. Subgroup analysis revealed that the correlation was more pronounced in middle-aged and young adults, females, and eutrophic individuals (interaction P value < 0.001).

**Conclusion:**

The TyG index can be a strong predictor of T2DM and is more useful for estimating the risk of T2DM in young and middle-aged adults, females, and eutrophic people.

## INTRODUCTION

Type 2 diabetes mellitus (T2DM) has become another important entity that threatens human health following cardiovascular and cerebrovascular diseases, tumors and chronic kidney disease ^(1)^. In the later stage of T2DM, multiple systems are involved, resulting in serious macrovascular and microvascular complications due to accelerated atherosclerosis ^(2)^. Therefore, it is extremely important to identify the related risk factors for T2DM to achieve early diagnosis and management of this condition.

Insulin resistance (IR) and β-cell dysfunction are indispensable mechanisms for the onset of T2DM. Some current investigations unanimously concluded that IR can function as an initial factor for the onset of T2DM and that the relationship between IR and T2DM affects β-cell dysfunction in the Chinese population ^(3)^. Consequently, assessing IR status is crucial for identifying people at high risk of developing T2DM. Currently, the euglycemic hyperinsulinemic clamp test (EHCT) is considered the gold standard test for the assessment of insulin resistance ^(4)^; however, it is less commonly applied in clinical and large-scale studies because of its complex operation. Fortunately, current studies have shown that the triglyceride-glucose (TyG) index, which is determined by fasting triglyceride (TG) and fasting blood glucose (FBG) levels, can be used to evaluate insulin resistance as well as the EHCT and homeostasis model assessment for insulin resistance (HOMA-IR) ^(5^,^6)^. Compared with the insulin index, the TyG index is more readily available in clinical practice and can be alternatively used as a reliable biomarker for assessing IR in many cardiovascular studies ^(7^,^8^,^9^,^10)^. It can also be used to predict the risk of adverse cardiovascular events in the T2DM population ^(11)^. Additionally, some epidemiological studies have shown that the TyG index is associated with the risk of T2DM ^(12^,^13)^. To the best of our knowledge, one 6-year follow-up investigation focused on the correlation between the TyG score and T2DM in the Chinese population, and it included only subjects with normal weight ^(14)^, which makes it difficult to apply to the general population in China. Although previous studies demonstrated that a high TyG index was positively correlated with the occurrence of T2DM, the relationship of T2DM with different TyG indices remains an important unsolved problem.

The current study was therefore designed to determine whether the TyG index is correlated with the incidence of T2DM on the basis of 5-year follow-up data obtained from the general population in China.

## SUBJECTS AND METHODS

### Study population

This was a retrospective cohort study with data collected from 9,720 residents in an urban community in Wuhu, Anhui, China. All participants underwent physical examination once a year from 2015 through 2020 at the First Affiliated Hospital of Wannan Medical College. The participants were excluded from the current study if they ^(1)^ had incomplete clinical and biochemical data; ^(2)^ had diabetes mellitus at baseline; ^(3)^ had recently used glucocorticoids or antipsychotics; or ^(4)^ had tumors, autoimmune disorders, or blood system conditions. After excluding 496 participants who had baseline T2DM and an additional 668 participants with missing data, we proceeded with our analysis. In total, 8576 subjects were ultimately included. The use of human information data does not cause bodily harm and does not involve sensitive personal information or commercial interests, which is in accordance with the 32^nd^ regulation of *Measures for Ethical Review of Life Sciences and Medical Research Involving Human Beings.* The study was exempt from ethical review.

### Data collection

The participants’ data were collected from annually repeated physical examinations via standardized spreadsheets. From 2015-2020, each participant participated in the physical examination at the same time yearly at the First Affiliated Hospital of Wannan Medical College, with a total of six physical examination data points. Blood pressure, height, and weight were measured by physicians via a medical apparatus. Data on age, sex, history of hypertension, smoking status and drinking status were collected by physicians through questionnaires. Blood samples were collected and analyzed by laboratory physicians.

The baseline demographic information included age and sex, and the baseline clinical and laboratory data consisted of systolic blood pressure (SBP); diastolic blood pressure (DBP); weight; height; body mass index (BMI); history of smoking, drinking and hypertension; absolute value of neutrophils (NEUT) and absolute value of lymphocytes (LYMPH); and red blood cell (RBC), hemoglobin (Hb), albumin (ALB), globulin (GLB), total protein (TP), aspartate transaminase (AST), alanine aminotransferase (ALT), fasting blood glucose (FBG), triglyceride-glucose (TyG) index, triglyceride (TG), total cholesterol (TC), high-density lipoprotein cholesterol (HDL-C), low-density lipoprotein cholesterol (LDL-C), uric acid (UA), and urea nitrogen (BUN) levels.

Blood pressure was measured while the participants were in a seated position with their arms supported at the level of their heart using mercury sphygmomanometers after at least 5 minutes of rest in a quiet room. SBP and DBP were defined as the average of both arm readings. Height was measured to the nearest 0.1 cm via a stadiometer, and weight was measured to the nearest 0.1 kg via a digital scale. BMI was calculated as weight (kg)/height squared (m^2^), and eutrophic individuals were defined as those with a BMI < 24 kg/m^2^ ^(15^,^16^,^17)^. Overweight subjects were defined as those with a BMI ≥ 24 and < 28 kg/m^2^. Obesity was defined as a BMI ≥ 28 kg/m^2^. The smoking status of the participants was divided into 2 categories: never smoker and current smoker (No/Yes). Drinking status among participants was divided into 2 categories: never drinker and current drinker (No/Yes). Blood samples were obtained from the antecubital vein after the participants had fasted overnight for at least 10 hours. The samples were sent to the clinical laboratory of the First Affiliated Hospital of Wannan Medical College.

Biochemistry tests, including tests of FBG, total cholesterol (TC), high-density lipoprotein cholesterol (HDL-C), and triglyceride (TG) levels, were performed via an automatic analyzer. The TyG index was quantified as Ln [fasting triglyceride (mg/dL) × fasting blood glucose (mg/dL)/2], as previously described ^(18)^.

### Outcomes

According to previous studies, the outcome was incident T2DM, which was defined as a fasting plasma glucose level ≥ 7 mmol/L or self-reported symptoms or laboratory findings suggestive of T2DM ^(19)^. The outcome was based on one measurement of fasting glucose.

### Statistical analysis

All the statistical analyses were performed via SPSS software version 21.0 (SPSS Inc., Chicago, IL) and GraphPad Prism 8.0 (San Diego, California, USA). Quantitative variables are presented as the mean values ± standard deviation (SD) or the medians (interquartile range) as appropriate. The Kolmogorov-Smirnov test was used to test normality. Baseline characteristics were compared between people with subsequent T2DM and those without subsequent T2DM. Comparisons of continuous variables between two groups were performed via the independent *t* test when the variables conformed to a normal distribution, and the Mann-Whitney U test was used when the variables conformed to nonnormal distributions. Categorical variables are expressed as the numbers and percentages, and Pearson’s chi-square test was used to evaluate the differences between two groups. The association between the TyG index and incident T2DM was determined via univariate and multivariate Cox proportional hazard models. The subjects were subsequently divided into three groups on the basis of the tertile of the TyG index, in which tertile 1 was defined as the reference group. Pearson’s chi-square test, the Kruskal-Wallis test, and one-way ANOVA were used to compare the baseline characteristics of all participants classified by tertiles of the TyG index. The associations between tertiles of the TyG index and incident T2DM were determined via univariate and multivariate Cox proportional hazard models. Model 1 was adjusted for age and sex, and Model 2 was adjusted for variables in Model 1 plus SBP, DBP, BMI, smoking status, history of hypertension, ALB, GLB, AST, ALT, HDL-C and BUN. The results are presented as hazard ratios (HRs) ± 95% confidence intervals (CIs), and statistical significance is indicated. Survival was calculated via Kaplan-Meier survival plots, and differences between distributions of survival were assessed via the log-rank test. Finally, stratified analyses were conducted on the basis of baseline age (<60 years and ≥ 60 years), sex, and BMI (<24 kg/m^2^, 24~28 kg/m^2^ and ≥ 28 kg/m^2^) to examine the consistency of the effect of the TyG index on the risk of T2DM. *P* values < 0.05 (two-tailed) were considered statistically significant.

## RESULTS

### Baseline characteristics of the T2DM and non-T2DM groups

Overall, 8576 participants were followed for five consecutive years in this study, among whom 882 developed T2DM and 7,694 did not within five years. Table 1 summarizes the baseline demographic, clinical, and laboratory characteristics of the two groups of participants. The participants were older and more often male in the T2DM group than in the group without T2DM (46.25 ± 13.70% *vs.* 57.06 ± 12.41%; 59.2% *vs.* 71.1%, respectively). The subjects in the T2DM group had higher levels of SBP and DBP, weight, BMI, GLB, NEUT and LYMPH, RBC, Hb, AST, ALT, FBG, TyG index, TG, TC, UA and BUN, as well as a longer history of hypertension, drinking and smoking, yet lower PLT, ALB and HDL-C levels. The differences in the aforementioned indicators were significant (all *P* <0.05), whereas there were no significant differences in TP, LDL-C or height between the two groups (*P* > 0.05).

**Table 1:** Baseline characteristics of participants by the presence of T2DM

	Without T2DM (n=7694)	T2DM (n=882)	*P* value
**Demographic characteristics**			
Age (years)	46.25±13.70	57.06±12.41	<0.001
Sex, n (%)			<0.001
Male	4552 (59.2%)	627 (71.1%)	
**Clinical characteristics**			
SBP (mmHg)	115.00 (105.00,125.00)	125.00 (115.00,135.00)	<0.001
DBP (mmHg)	75.00 (70.00,80.00)	80.00 (70.00,85.00)	<0.001
Height (cm)	165.43±8.34	165.03±8.34	0.170
Weight (kg)	65.96±12.31	69.22±12.32	<0.001
BMI (kg/m^2^)	23.63±3.39	25.32±3.49	<0.001
Smoking, n (%)			<0.001
No	6039 (78.4%)	643 (72.9%)	
Yes	1655 (21.6%)	239 (27.1%)	
Drinking, n (%)			0.008
No	5452 (70.8%)	587 (66.6%)	
Yes	2242 (29.2%)	295 (33.4%)	
Hypertension, n (%)	1009 (13.1%)	353 (40.0%)	<0.001
**Laboratory characteristics**			
NEUT (10^9^ /L)	3.55±1.22	3.87±116	<0.001
LYMPH (10^9^ /L)	2.05±0.58	218±0.71	<0.001
RBC (10^12^/L)	4.65±0.48	4.70±0.46	0.001
PLT (10^12^/L)	188.53±55.91	180.92±58.30	0.001
Hb (g/L)	140.99±15.20	143.86±1415	<0.001
ALB (g/L)	47.08±3.35	46.73±3.33	0.003
GLB (g/L)	27.38±3.65	27.75±3.95	0.004
TP (g/L)	74.46±4.00	74.48±4.21	0.906
AST (U/L)	19.00 (15.00,23.00)	20.00 (16.00,25.00)	<0.001
ALT (U/L)	20.00 (14.00,30.00)	24.00 (17.00,37.00)	<0.001
FPG (mmol/L)	5.27±0.58	7.35±1.96	<0.001
Ty G	8.67±0.58	9.29±0.65	<0.001
LDL-C (mmol/L)	2.34±0.89	2.38±0.93	0.243
HDL-C (mmol/L)	1.52±0.38	1.39±0.33	<0.001
TG (mmol/L)	1.34 (0.94,1.99)	1.83 (1.27,2.78)	<0.001
TC (mmol/L)	4.61±0.98	4.79±1.03	<0.001
UA (µmol/L)	332.21±8518	35718±85.57	<0.001
BUN (mg/dL)	5.38±1.44	5.76±1.45	<0.001

ALB: albumin; ALT: alanine aminotransferase; AST: aspartate transaminase; BMI: body mass index; BUN: blood urea nitrogen; DBP: diastolic blood pressure; FBG: fasting blood glucose; GLB: globulin; Hb: hemoglobin; HDL-C: high-density lipoprotein; LDL-C: low-density lipoprotein cholesterol; LYMPH: lymphocyte; NEUT: neutrophils; PLT: platelet; RBC: red blood corpuscle; SBP: systolic blood pressure; TC: total cholesterol; T2DM: type 2 diabetes mellitus; TG: triglyceride; TP: total protein; TyG: triglyceride-glucose; UA: uric acid.

### TyG index and T2DM

Univariate and multivariate analyses between variables and the occurrence of T2DM are presented in Table 2. Univariate Cox regression analysis revealed that age, sex, SBP, DBP, weight, BMI, smoking status, history of hypertension, ALB, GLB, AST, ALT, FBG, TyG index, TG, HDL-C and BUN were associated with the incidence of T2DM and that TyG (HR: 3.348; 95% CI: 3.004-3.731, *P* < 0.001), age (HR: 1.042; 95% CI: 1.036-1.049, *P* < 0.001), SBP (HR: 1.010; 95% CI: 1.004-1.016, *P* = 0.001), BMI (HR: 1.034; 95% CI: 1.012-1.056, *P* = 0.002), history of hypertension (HR: 1.496; 95% CI: 1.281-1.747, *P* < 0.001), and ALT (HR: 1.010; 95% CI: 1.006-1.014, *P* < 0.001) were independent risk factors for T2DM. On the basis of the data generated from the above analyses, we developed a Cox proportional risk model fitted with robust estimators and used TyG as a continuous covariate to assess the risk of incidence of T2DM in all participants. Model 1 was adjusted for age and sex. The corrected covariates for Model 2 included age; sex; SBP; DBP; BMI; smoking status; history of hypertension; and the levels of GLB, ALB, AST, ALT, HDL-C and BUN. According to both models, the TyG index was significantly and independently associated with the incidence of T2DM (*P* < 0.001) (Table 3).

**Table 2:** Results of univariate and multivariate analysis and predictors of incidence of T2DM

	Univariate analysis		Multivariate analysis	
	HR (95% CI)	*P* value	HR (95% CI)	*P* value
Age (years)	1.050 (1.045-1.054)	<0.001	1.042 (1.036-1.049)	<0.001
Sex (male)	0.609 (0.527-0.704)	<0.001	0.708 (0.530-0.714)	0.001
SBP (mmHg)	1.036 (1.032-1.040)	<0.001	1.010 (1.004-1.016)	0.001
DBP (mmHg)	1.037 (1.029-1.044)	<0.001	0.991 (0.981-1.000)	0.052
Weight (kg)	1.023 (1.018-1.027)	<0.001		
BMI (kg/m^2^)	1.123 (1.104-1.141)	<0.001	1.034 (1.012-1.056)	0.002
Smoking (No)	1.317 (1.135-1.528)	<0.001	1.003 (0.848-1.186)	0.972
Hypertension	3.816 (3.335-4.367)	<0.001	1.496 (1.281-1.747)	<0.001
ALB (g/L)	0.970 (0.951-0.990)	0.003	0.981 (0.960-1.004)	0.102
GLB (g/L)	1.026 (1.008-1.044)	0.004	0.974 (0.956-0.993)	0.008
AST (U/L)	1.009(1.006-1.011)	<0.001	0.984 (0.974-0.994)	0.002
ALT (U/L)	1.005 (1.004-1.006)	<0.001	1.010 (1.006-1.014)	<0.001
FPG (mmol/L)	1.556 (1.530-1.582)	<0.001		
Ty G	3.633 (3.326-3.976)	<0.001	3.348 (3.004-3.731)	<0.001
HDL-C (mmol/L)	0.345 (0.280-0.424)	<0.001	1.008 (0.807-1.260)	0.942
TG (mmol/L)	1.249 (1.213-1.286)	<0.001		
BUN (mg/dL)	1.156 (1.115-1.199)	<0.001	1.015 (0.968-1.064)	0.544

ALB: albumin; ALT: alanine aminotransferase; AST: aspartate transaminase; BMI: body mass index; BUN: blood urea nitrogen; DBP: diastolic blood pressure; FBG: fasting blood glucose; GLB: globulin; HDL-C: high-density lipoprotein; SBP: systolic blood pressure; T2DM: type 2 diabetes mellitus; TG: triglyceride; TyG: triglyceride-glucose.

**Table 3:** Multivariable-adjust HRs and 95%CI of the TyG index on incidence of T2DM

TyG index		Unadjusted HR (95% CI)	Model 1 HR (95% CI)	Model 2 HR (95% CI)
Overall TyG	Continuous per unit increase	3.633 (3.326-3.976)	3.609 (3.286-3.964)	3.348 (3.004-3.731)
	*P* value	<0.001	<0.001	<0.001

Data presented were HRs and 95% CIs.

Model 1: adjust for age and sex.

Model 2: adjust for age, sex, SBP, DBP, BMI, smoking, history of hypertension, ALB, GLB, AST, ALT, HDL-C, BUN.

ALB: albumin; ALT: alanine aminotransferase; AST: aspartate transaminase; BMI: body mass index; BUN: blood urea nitrogen; DBP: diastolic blood pressure; GLB: globulin; HDL-C: high-density lipoprotein; SBP: systolic blood pressure; T2DM: type 2 diabetes mellitus; TyG: triglyceride-glucose.

### Baseline characteristics according to tertile of the TyG index

To better understand the relationship between the TyG index and T2DM, we categorized the TyG level into three groups (Tertile 1 group: *n* = 2859, TyG index ≤ 8.4328; Tertile 2 group: *n* = 2859, 8.4329 ≤ TyG index < 8.9608; Tertile 3 group: *n* = 2858, 8.9609 ≤ TyG index < 11.9178). The average TyG values in the three groups were 8.15 (7.93, 8.30), 8.68 (8.56, 8.83), and 9.31 (9.11, 9.63), respectively. Five-year follow-up revealed that 61 participants (2.1%) in the tertile 1 group, 226 (7.9%) in the tertile 2 group, and 595 (20.9%) in the tertile 3 group developed T2DM. Furthermore, we observed significant differences in age, sex, SBP, DBP, height, weight, BMI, history of smoking and drinking and hypertension, NEUT, LYMPH, RBC, Hb, ALB, GLB, TP, AST, ALT, FBG, TG, LDL-C, HDL-C, TC, UA, and BUN among the three groups (all *P* < 0.05) (Table 4).

**Table 4:** Baseline characteristics of participants by TyG index

	Tertile 1 (n=2859)	Tertile 2 (n=2859)	Tertile 3 (n=2858)	*P* value
**Demographic characteristics**				
Age (years)	44.11±13.82	48.36±14.31	49.62±13.14	<0.001
Sex, n (%)				<0.001
Male	1309 (45.8%)	1757 (61.5%)	2113 (73.9%)	
**Clinical characteristics**				
SBP (mmHg)	112.18±13.66	117.49±14.84	121.25±14.83	<0.001
DBP (mmHg)	73.63±8.29	76.57±8.62	79.47±8.85	<0.001
Height (cm)	164.29±8.31	165.34±8.39	166.54±8.18	<0.001
Weight (kg)	60.79±11.56	65.34±11.51	70.07±12.28	<0.001
BMI (kg/m^2^)	22.43±3.21	23.81±3.17	25.17±3.37	<0.001
Smoking, n (%)				<0.001
No	2453 (85.8%)	2224 (77.8%)	2005 (70.1%)	
Yes	406 (14.2%)	635 (22.2%)	853 (29.9%)	
Drinking, n (%)				<0.001
No	2293 (80.2%)	2001 (70.0%)	1745 (61.1%)	
Yes	566 (19.8%)	858 (30.0%)	1113 (38.9%)	
Hypertensin, n (%)	244 (8.5%)	442 (15.5%)	676 (23.7%)	<0.001
**Laboratory findings**				
NEUT (10^9^/L)	3.32±1.17	3.57±1.17	3.87±1.25	<0.001
LYMPH (10^9^/L)	1.93±0.54	2.06±0.61	2.20±0.61	<0.001
RBC (10^12^/L)	4.52±0.45	4.67±0.47	4.77±0.47	<0.001
PLT (10^12^/L)	189.46±55.18	187.79±5731	185.99±56.08	0.066
Hb (g/L)	13632±14.79	141.45±14.96	146.09±14.01	<0.001
ALB (g/L)	46.75±3.24	47.04±3.28	47.35±3.47	<0.001
GLB (g/L)	27.15±3.57	27.37±3.63	27.73±3.80	<0.001
TP (g/L)	73.90±3.95	74.41±3.89	75.08±4.12	<0.001
AST (U/L)	18.84±8.10	20.40±11.64	22.57±10.62	<0.001
ALT (U/L)	16.00 (12.00,23.00)	20.00 (14.00,29.00)	26.00 (18.00,40.00)	<0.001
FBG (mmol/L)	5.12±0.54	5.40±0.73	5.96±1.45	<0.001
Ty G	8.15 (7.93,8.30)	8.68 (8.56,8.83)	9.31 (9.11,9.63)	<0.001
LDL-C (mmol/L)	2.29±0.82	2.47±0.87	2.28±0.96	<0.001
HDL-C (mmol/L)	1.66±0.39	1.51±0.34	1.37±0.33	<0.001
TG (mmol/L)	0.83±0.20	1.42±0.26	2.93±1.53	<0.001
TC (mmol/L)	4.33±0.88	4.62±0.93	4.94±1.04	<0.001
UA (µmol/L)	298.36±73.87	332.90±79.67	373.09±85.79	<0.001
BUN (mg/dL)	5.29±1.51	5.46±1.44	5.50±1.37	<0.001
Incident T2DM, n (%)	61 (2.1%)	226 (7.9%)	595 (20.8%)	<0.001

ALB: albumin; ALT: alanine aminotransferase; AST: aspartate transaminase; BMI: body mass index; BUN: blood urea nitrogen; DBP: diastolic blood pressure; FBG: fasting blood glucose; GLB: globulin; Hb: hemoglobin; HDL-C: high-density lipoprotein; LDL-C: low-density lipoprotein cholesterol; LYMPH: lymphocyte; NEUT: neutrophils; PLT: platelet; RBC: red blood corpuscle; SBP: systolic blood pressure; TC: total cholesterol; T2DM: type 2 diabetes mellitus; TG: triglyceride; TP: total protein; TyG: triglyceride-glucose; UA: uric acid.

### Tertiles of the TyG index and T2DM

**Figure 1:**
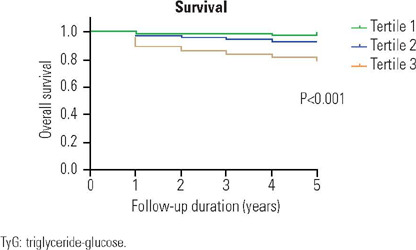
Kaplan-Meier survival curve by TyG index tertiles.

To observe the relationship between the TyG index and T2DM in the three groups, we established a Cox proportional hazard regression model (Table 5), with the data in the tertile 1 group serving as the control, to analyze the risk of developing T2DM in tertiles 2 and 3. After adjusting for age, sex, SBP, DBP, BMI, smoking status, history of hypertension, ALB, GLB, AST, ALT, HDL-C and BUN, the risk of developing T2DM increased by 2.706 times (HR: 2.706, 95% CI: 2.033-3.602, *P* < 0.001) in the tertile 2 group and by 6.412 times (HR: 6.412, 95% CI: 4.869-8.443, *P* < 0.001) in the tertile 3 group with every added 1- index. A Kaplan-Meier plot was used to compare the cumulative incidence of T2DM in the three groups (Figure 1). The results indicated that the risk of T2DM incidence was greater in the tertile 3 group than in the tertile 2 group, and the cumulative incidence of T2DM increased gradually across the three groups (log rank *P* < 0.001).

**Table 5:** Univariate and multivariate Cox analyses of T2DM in tri-sectional TyG groups

	Non-adjusted HR (95% CI)	Model 1			Model 2	
		*P* value	HR (95% CI)	*P* value	HR (95% CI)	*P* value
Tertile 1	Ref.		Ref.		Ref.	
Tertile 2	3.778 (2.847-5.013)	<0.001	3.083 (2.323-4.093)	<0.001	2.706 (2.033-3.602)	<0.001
Tertile 3	10.560 (8.114-13.744)	<0.001	8.437 (6.477-10.990)	<0.001	6.412 (4.869-8.443)	<0.001

Model 1: adjust for age and sex

Model 2: adjust for age, sex, SBP, DBP, BMI, smoking, history of hypertension, ALB, GLB, AST, ALT, HDL-C, BUN.

ALB: albumin; ALT: alanine aminotransferase; AST: aspartate transaminase; BMI: body mass index; BUN: blood urea nitrogen; DBP: diastolic blood pressure; GLB: globulin; HDL-C: high-density lipoprotein; SBP: systolic blood pressure; T2DM: type 2 diabetes mellitus; TyG: triglyceride-glucose.

TyG: triglyceride-glucose.

### Subgroup analysis and interaction test

An evaluation of the interactions of age, sex and BMI with the TyG index and incidence of T2DM is shown in Table 6. By stratifying the above indicators, young adults, females, and individuals with a BMI < 24 kg/m^2^ were at a significantly greater risk of developing TyG-related diabetes than were their counterparts (*P* interaction < 0.001). In terms of age stratification, the risk of TyG index-related diabetes was significantly greater in middle-aged and young adults than in aged adults [HR (increase per SD): less than 60 years old: 3.190 *vs*. ≥ 60 years old: 3.149]. Sex was stratified, and the results revealed that the risk of TyG index-related diabetes was greater in females than in males [HR (increase per SD): female: 3.870 *vs*. male: 3.197]. Stratifying BMI demonstrated that the risk of TyG index-related diabetes was greater in nonobese people than in overweight and obese people [HR (per SD increase): BMI < 24 kg/m^2^: 3.522 *vs*. BMI ≥ 24, < 28 kg/m^2^: 3.423, BMI ≥ 28 kg/m^2^: 2.815] (all *P* < 0.05).

**Table 6:** Stratified association between TyG index and T2DM by age, sex and BMI

Variables	Unadjusted HR (95% CI)	*P* value	Model 1 HR (95% CI)	*P* value	Model 2 HR (95% CI)	*P* value	*P* interaction
Age							<0.001
≥60 years	4.024 (3.596-4.502)	<0.001	3.438 (3.043-3.884)	<0.001	3.190 (2.664-3.819)	<0.001	
≥60 years	3.313 (2.835-3.872)	<0.001	3.398 (2.903-3.978)	<0.001	3.149 (2.731-3.631)	<0.001	
Sex							<0.001
Male	3.116 (2.795-3.472)	<0.001	3.374 (3.021-3.769)	<0.001	3.197 (2.814-3.632)	<0.001	
Female	5.214 (4.421-6.148)	<0.001	4.237 (3.519-5.102)	<0.001	3.870 (3.137-4.776)	<0.001	
BMI							<0.001
<24 kg/m^2^	4.076 (3.500-4.746)	<0.001	3.741 (3.187-4.393)	<0.001	3.522 (2.977-4.166)	<0.001	
24~28 kg m^2^	3.309 (2.869-3.815)	<0.001	3.464 (2.989-4.014)	<0.001	3.423 (2.865-4.089)	<0.001	
≥28 kg/m^2^	2.505 (2.031-3.090)	<0.001	2.673 (2.141-3.336)	<0.001	2.815 (2.199-3.604)	<0.001	

Model 1: adjust for age and sex.

Model 2: adjust for age, sex, SBP, DBP, BMI, smoking, history of hypertension, ALB, GLB, AST, ALT, HDL-C, BUN.

ALB: albumin; ALT: alanine aminotransferase; AST: aspartate transaminase; BMI: body mass index; BUN: blood urea nitrogen; DBP: diastolic blood pressure; GLB: globulin; HDL-C: high-density lipoprotein; SBP: systolic blood pressure; T2DM: type 2 diabetes mellitus; TyG: triglyceride-glucose.

## DISCUSSION

Insulin resistance serves as an important underlying cause for the development of T2DM, and the triglyceride glucose (TyG) index represents an easily accessible surrogate biomarker for the determination of insulin resistance. To evaluate the correlation of the TyG index with the risk of developing T2DM, we conducted a retrospective cohort study in a large population and found that the TyG index was consistently stable across the statistical models and independently associated with T2DM incidence. Compared with the study subjects with the lowest TyG index quantile, those with the highest TyG index had a 6.412-fold greater risk of developing T2DM. Further subgroup analysis revealed that the TyG index was more strongly associated with T2DM risk in young and middle-aged adults, females, and eutrophic individuals (BMI < 24 kg/m^2^).

T2DM is a metabolic disorder characterized by islet beta-cell dysfunction and insulin resistance (IR), which are the major factors associated with this disease ^(3)^. IR is defined as the reduced efficiency of insulin in promoting glucose uptake and glucose utilization and can promote the progression of diabetes by inducing an imbalance in glucose metabolism, altering lipid metabolism in the whole body and causing endothelial dysfunction ^(20)^. The indicators used to evaluate IR include the hyperinsulinemic-glucose clamp and HOMA-IR, which are less commonly used in routine laboratory studies in the clinic because of their complexity of measurement. A previous study demonstrated that the TyG index can serve as an alternative indicator for estimating IR because of its simple performance and cost-effectiveness ^(21)^. The TyG index is a combination of triglycerides (TGs) and fasting blood glucose (FBG). The level of the latter mainly reflects IR status from the liver, whereas the TG level chiefly indicates IR from fat cells ^(22)^. Studies have also shown that the TyG index is an important prognostic indicator for patients with prediabetes and can predict the occurrence of cardiovascular and cerebrovascular adverse events ^(21)^. Therefore, the TyG index can be competent in predicting the occurrence of T2DM.

A study revealed that the risk of T2DM increased with increasing TyG index among the rural Chinese population during follow-up for 3.1 years ^(23)^, which involved only the rural population over 75 years of age. However, the relationship between the TyG index and T2DM in people between 18 and 85 years of age remains unclear, since the age of onset of T2DM tends to be younger ^(24)^; in particular, a greater incidence of T2DM is observed in the population aged 30-55 years. To observe the homogeneity of the TyG index in early diagnosis for this population, we subgrouped our participants and found that the correlation between the TyG index and T2DM incidence was greater in subjects aged 1,860 years than in those aged over 60 years. Recent studies have demonstrated that young patients diagnosed with diabetes have a greater risk of cardiovascular disease and death than do elderly patients ^(25)^, which indicates that the TyG index may be useful for the early identification of individuals at risk of T2DM ^(26)^. Our observations revealed a stronger correlation between the TyG index and T2DM in young individuals, suggesting that our findings can be beneficial for preventing T2DM at an early stage in this population.

Stratifying by sex, we found that the independent correlation between the TyG index and T2DM incidence was greater in females than in males. This is probably associated with the fact that free fatty acids, which are considered to be major factors in the occurrence of insulin resistance, are apt to be stored in females ^(27^,^28)^. In addition, other studies have shown that sex hormone binding globulin (SHBG) is closely related to systemic metabolism and that SHBG can reflect a variety of circulating lipid and metabolite changes related to insulin resistance ^(29)^. Furthermore, SHBG has been reported to affect changes in estrogen concentration, and in women, subphysiological and physiological estrogen concentrations are related to an increased incidence of T2DM, which may explain the difference in insulin resistance between men and women ^(30)^. Moreover, we found that the TyG index can be used to predict the onset of T2DM in eutrophic individuals. This may be associated with the significant fat distribution and lipodystrophy in the eutrophic population ^(31^,^32)^.

Our findings also have several other clinical implications. Tables 1 and 2 show that smoking is positively associated with the incidence of T2DM. Studies have shown that people who smoke have twice the risk of impaired insulin secretion than people who never smoke ^(33)^. Nicotine has been linked to an increased risk of T2DM from smoking. Nicotine affects insulin secretion through nicotinic acetylcholine receptors on islet beta cells and mediates nicotine to increase the apoptosis of islet beta cells through the mitochondrial receptor pathway ^(34)^.

The occurrence of T2DM is influenced by both a history of hypertension and a family history of diabetes. Our study revealed a clear association between a prior diagnosis of hypertension and the likelihood of developing T2DM. A prospective cohort study conducted in the United States revealed that hypertensive patients have a 2.5-fold increased risk of developing T2DM compared with the general population ^(35)^. Consequently, maintaining strict control of blood pressure among individuals with diabetes is crucial to significantly reduce diabetes-related mortality and complications ^(36)^. Furthermore, numerous studies have established a correlation between having a familial predisposition to diabetes and a heightened vulnerability to developing T2DM. According to previous research, individuals with a family history of diabetes exhibit diminished insulin production, which subsequently elevates their risk of T2DM ^(37)^. The underlying connection between a positive family history and T2DM can be attributed to shared environmental and genetic factors that influence behavior, lifestyle choices, and metabolic processes ^(38)^.

In summary, we validated that the TyG index is a strong predictor of T2DM, and the TyG index generated from our subgroup analysis demonstrated greater sensitivity in predicting the risk of T2DM in the younger group, the female population and the eutrophic population. These findings suggest that the TyG index is beneficial for the early identification of individuals at risk of T2DM, particularly those in the aforementioned population groups. Additionally, the TyG index appears to be a predictor that is superior to fasting plasma glucose or triglycerides for the potential development of T2DM in normoglycemic patients. The early detection of targeted interventions in individuals predisposed to T2DM holds significant potential to significantly alleviate the overall burden of the disease within the population. By leveraging a comprehensive assessment that incorporates smoking history, familial predisposition to diabetes, history of hypertension, and other established risk factors, we can effectively identify and stratify individuals at heightened risk, thereby facilitating crucial advancements in the prevention of T2DM.

There are several limitations to this study due to the nature of the current observations. Although we carefully adjusted for known and suspected risk factors, we cannot rule out the possibility of residual confounders. For example, we did not consider the effect of lipid-lowering drugs, which may be a potential risk factor for T2DM and may have residual confounding effects. We lacked 2-hour oral glucose tolerance tests and HBA1c tests to diagnose T2DM, and a diagnosis of diabetes on the basis of a single fasting glucose measurement may have resulted in an underestimation of T2DM incidence. In addition, this study was based on the Chinese population, and whether the findings are generalizable to other ethnicities needs to be verified.

In conclusion, this study revealed a significant correlation between the TyG index and T2DM and demonstrated that the TyG index is an independent predictor for estimating the risk of and early screening for T2DM. Moreover, the TyG index seems to be more strongly associated with T2DM risk in young and middle-aged adults, females, and eutrophic individuals.
